# Development of Interactional Synchrony: Examining the Role of Maternal Emotion Dysregulation

**DOI:** 10.3390/bs16081326

**Published:** 2026-08-03

**Authors:** Jessica T. Kelly, Ellen A. Knowles, Jessy L. Thomas, Emily Hermann, McKensey Berg, Patti Berg-Poppe, Whitney Lucas Molitor, Matthew A. Barker, Staci Wietfeld, BreAnne A. Danzi

**Affiliations:** 1 Department of Psychology, University of South Dakota, 414 East Clark Street, Vermillion, SD 57069, USA; jessica.kelly@coyotes.usd.edu (J.T.K.); ellen.knowles@coyotes.usd.edu (E.A.K.); jessy.thomas@coyotes.usd.edu (J.L.T.); emily.hermann@coyotes.usd.edu (E.H.); mckensey.berg@coyotes.usd.edu (M.B.); 2 Department of Physical Therapy, University of South Dakota, 414 East Clark Street, Vermillion, SD 57069, USA; patti.berg@usd.edu (P.B.-P.);; 3 Department of Occupational Therapy, University of South Dakota, 414 East Clark Street, Vermillion, SD 57069, USA; whitney.lucasmolitor@usd.edu; 4 Avera McKennan Health Systems, 1325 S Cliff Avenue, Sioux Falls, SD 57105, USA; 5 School of Medicine, University of South Dakota, 1400 W. 22nd Street, Sioux Falls, SD 57105, USA

**Keywords:** interactional synchrony, maternal emotional dysregulation, mother–infant dyads

## Abstract

Infancy is a period of rapid development, with interactional synchrony between mother and infant being one of the earliest bonding experiences. However, little research has characterized developmental shifts in mother–infant interactional synchrony. Maternal emotion dysregulation may influence interactional synchrony, yet little research has evaluated the impact of maternal emotion dysregulation on synchrony over time. This project aims to (1) evaluate changes in mother–infant interactional synchrony over time, (2) assess the relationship between maternal emotion dysregulation and mother–infant interactional synchrony, and (3) evaluate the impact of maternal emotion dysregulation on interactional synchrony over time. Participants were 90 mother–infant dyads who had a vaginal delivery. Mothers reported on their functioning and behavioral observation data were collected at two time points (4 and 6 months after birth) and coded for markers of synchrony, including physical proximity, visual orientation, upper-body orientation, and dyadic involvement. Overall synchrony, upper-body orientation, and dyadic involvement increased from 4 to 6 months. Maternal emotion dysregulation at 4 months did not predict any markers of synchrony at 4 months, nor did it predict any markers of synchrony over time (6 months). These findings offer insights into early developmental changes in synchrony and suggest that these synchrony markers may be robust to variations in maternal emotional regulation.

## 1. Introduction

Mother–infant interactional synchrony refers to the coordinated, dynamic, and reciprocal interaction between the mother and infant marked by mutual responsiveness and social reward (Reyna & Pickler, 2009; Freeman, 2024). Interactional synchrony has been implicated in supporting attachment development and long-term infant psychological and social development (Bell, 2020; Leclère et al., 2014). Furthermore, the relationship between mother and infant develops and becomes increasingly complex as infants learn to process cues from others rapidly (Feldman, 2012a, 2012b). However, current understanding of how interactional synchrony develops during infancy has been inconsistent and contradictory (Northrup & Iverson, 2020; Provenzi et al., 2018).

Within the existing literature base, numerous infant- and mother-driven factors have been posited to influence the synchronous relationship between mother and infant; however, the notion that the mother’s emotional functioning may influence interactional synchrony has been a particular area of interest among researchers (Lotzin et al., 2016). Indeed, women often experience heightened emotionality during pregnancy and the postpartum period due to increased life stressors and biological changes (Bjelica et al., 2018). Some research has suggested that infants are sensitive to their mothers’ emotional states (E. Z. Tronick, 1989); this would suggest that mothers’ emotional regulation may have a measurable impact on mother–infant interactional synchrony.

Considering inconsistent understandings of the development of mother–infant interactional synchrony, the current study aims to characterize the developmental changes of markers of interactional synchrony during infancy. Moreover, the current study aims to evaluate how mothers’ emotional dysregulation influences interactional synchrony in mother–infant dyads. Given that the synchronous relationship develops rapidly, the current study aims to examine how mothers’ emotional dysregulation influences interactional synchrony over time.

### 1.1. Attachment Theory

Pioneered by John Bowlby and Mary Ainsworth, attachment theory has long served as a foundation for understanding the complex relationship between mothers and infants, as well as the infant’s development of independent regulation (Ainsworth, 1978; Bowlby, 1969; Bretherton, 1992). Similarly, there are known implications for disruptions in the mother–infant relationship (e.g., due to separation or loss; Ainsworth, 1978; Bowlby, 1969; Bretherton, 1992). For instance, strong mother–infant bonds are associated with infants’ feelings of safety in themselves and others, whereas weaker bonds are linked to greater anxiety toward the world and others (Ainsworth, 1978; Bowlby, 1969; Bretherton, 1992). Underlying this framework are complex developmental processes, characterized by micro moment-to-moment interactions between mothers and their infants, which serve as “building blocks” for what later becomes attachment.

### 1.2. Interactional Synchrony

Mother–infant interactional synchrony has been characterized and operationalized through several specific behavioral interactions between mothers and infants, including gaze (Doba et al., 2022; Granat et al., 2017; Lotzin et al., 2015), affect (Feldman et al., 1999; Markova et al., 2020), facial affect (Kudinova et al., 2019; Lotzin et al., 2016), touch (Doba et al., 2022; Granat et al., 2017), and vocalization (Bell, 2020; Doba et al., 2022). Beyond individual behaviors, interactional synchrony has also been characterized by more complex or combinations of behaviors. For example, De Mendonça and colleagues (2011) also include the dyad involvement during play interaction as an index of the degree of mutual engagement in an activity, which may include object manipulation and joint attention.

The process of interactional synchrony between mother and infant can be understood as a specific component of the broader complex of mother–infant social interaction that is necessary for, and plays a significant role in, the long-term development of the mother–infant relationship (Provenzi et al., 2018). In addition to interactional synchrony, other dyadic processes identified in the literature include attunement, coordination, contingency, matching, reparation, and mirroring (Provenzi et al., 2018). All of these processes have been suggested to contribute to the quality of mother–infant interactions and infant developmental outcomes (Provenzi et al., 2018). A prominent study by Isabella and Belsky (1991) evaluated relationships in mother–infant dyads and found that greater mother–infant interactional synchrony was associated with secure attachment, whereas lower interactional synchrony was associated with insecure attachment style among dyads.

In sum, the ongoing give-and-take necessary for synchronous interactions, and the cumulative effects of these interactions between infant and mother, serve to lay a foundation for mother–child relationships and provide the framework for the development of attachment (Bell, 2020; Leclère et al., 2014). Furthermore, interactional synchrony has critical implications for the social, biological, and psychological development and functioning of the infant across the lifespan. Mother–infant interactional synchrony may promote attentional capabilities, self-regulation, and social and emotional functioning (Black & Logan, 1995; Feldman, 2007; Harrist et al., 1994; MacLean et al., 2014; Niedźwiecka et al., 2018).

#### The Development of Interactional Synchrony

Given the rate at which infants develop cognitively and socially (Hodel, 2018), mother–infant interactional synchrony likely changes in line with major developmental stages, yet research findings in this area are inconsistent (Northrup & Iverson, 2020; Provenzi et al., 2018). Many researchers have suggested that the earliest signs of synchronous behavior between mother and infant can be observed around 3 months of age, when aspects of facial interactions, vocal tone, and touch are identified as the primary forms of synchrony (Feldman, 2012b; Harel et al., 2011; E. Tronick et al., 1980). However, others have suggested that synchrony can be observed even earlier (Rubin, 1976).

Researchers have generally found that mutual eye gaze between mother and infant tends to decrease with age while shared attention to an activity may increase as infants get older (Bakeman & Adamson, 1984; Kaye & Fogel, 1980; Northrup & Iverson, 2020). Such a finding would support the notion that older infants are developmentally capable of engaging in activities that require more advanced processes. However, some studies have reported contrasting outcomes, suggesting that mother–infant shared attention declines with infant age (De Barbaro et al., 2016). Synchrony in vocalizations between mother and infant has been observed in dyads around 3 to 4 months of age (Beebe et al., 1988; Northrup & Iverson, 2020; Van Egeren et al., 2001). Some research has suggested that vocal synchrony tends to decrease as infants age (Hilbrink et al., 2015; Jaffe et al., 2001); however, other studies have found no significant directional change in mother–infant vocal synchrony over time (Northrup & Iverson, 2020). Dyadic involvement, the process by which mothers and infants mutually engage in an activity, has been examined in several studies; however, developmental changes in dyadic involvement, if any, remain unknown. Most research evaluating this construct has focused on toddlers (De Mendonça et al., 2011, 2019).

Motor synchrony between an infant and their mother is one of the few synchronous behaviors that has been evaluated longitudinally to investigate its developmental trajectory. Doi and colleagues (2011) assessed motor synchrony between infants and their mothers from ages 4 to 18 months and found an increase in motor synchrony over time (Doi et al., 2011). In general, physical proximity between an infant and mother tends to decrease with age as development allows for more complex movement and increased curiosity about the environment (Chen et al., 2023; Negayama & Trevarthen, 2022; Rheingold & Eckerman, 1970). Body orientation has also been evaluated in toddlers, supporting its usefulness as an indicator of synchrony across early developmental periods (De Mendonça et al., 2011, 2019). Despite the lack of consensus on how synchrony changes during infancy, an infant’s ability to engage in synchronous behaviors is influenced by typical developmental progress (Bell, 2020).

Findings on the influence of infants’ age on the synchronous relationship between mothers and infants are inconsistent, a limitation repeatedly noted in recent literature (Northrup & Iverson, 2020; Provenzi et al., 2018). Yet available research in this area suggests a significant and rapid shift in the development of mother–infant interactional synchrony, up to approximately 6 months of age (Feldman, 2012b; Hodel, 2018). Additionally, while some behaviors have been used more frequently to evaluate synchrony (e.g., gaze, vocalization), others have been studied to a lesser extent (e.g., body orientation). The current project evaluates both extensively studied and less-studied interactive behaviors in assessing mother–infant interactional synchrony.

### 1.3. Measuring Mother–Infant Interactional Synchrony

Despite years of research, interactional synchrony is often operationalized differently across the literature. Interactional synchrony has been evaluated using diverse methodologies (Baker & McGrath, 2011), which likely contribute to the long-standing disagreement over its definition. Specifically, interactional synchrony has been operationalized through simple behavioral markers (i.e., gaze, visual orientation; De Mendonça et al., 2011) or biobehavioral markers (i.e., heart rate, neural activity, oxytocin; Feldman, 2012a, 2012b). Indeed, the process of interactional synchrony has also been measured by prioritizing the degree of responsiveness within the mother–infant dyad or the timing of coordinated behaviors between dyads (Feldman, 2007, 2012a; Isabella & Belsky, 1991; Reyna & Pickler, 2009). Indeed, these operational differences have necessitated vastly different methodologies to evaluate interactional synchrony (i.e., biobehavioral operational definitions require more complex instruments compared to observation of simple behaviors).

Behavioral observations of mother–infant interactions have become a common method for evaluating interactional synchrony, and will be the primary method used in this study (Falk & Heckman, 2009). However, behavioral observations used to evaluate mother–infant synchrony have most commonly been conducted in laboratory settings (Provenzi et al., 2018). These laboratory settings often limit observations because they restrict typical mother–infant interactions (Falk & Heckman, 2009). The scarce literature on the developmental trajectory of interactional synchrony during infancy, along with inconsistent definitions, and the use of multiple behaviors to characterize it, suggests a substantial gap in understanding its complex nature.

### 1.4. Maternal Emotion Regulation and Mother–Infant Interactional Synchrony

Researchers studying emotion regulation in the context of parenthood argue that parents’ emotion-regulation abilities likely play a significant role in shaping their interactions with their infants and their child’s ongoing development (Rutherford et al., 2015). For instance, parental emotion regulation may influence early stages of infants’ emotional development, including coping skills (Zimmer-Gembeck et al., 2022). However, understanding emotion regulation in the context of pregnancy and motherhood is complex because several biological and social factors experienced by mothers significantly alter emotion-regulation abilities (Bjelica et al., 2018).

Maternal emotion dysregulation has been linked to several adverse implications for infant functioning across all major developmental stages. For instance, in infancy, maternal emotion dysregulation has been linked to general negative affect (Edwards et al., 2017) as well as impaired infant development of effective emotion regulation (Cassidy, 1994) and mother–infant attachment (Brake et al., 2020). When considering the role of maternal emotion regulation in supporting infant development, it is important to understand whether emotion dysregulation might negatively affect the mother–infant relationship. Furthermore, because mother–infant synchrony is a foundational process for the development of critical functions in infancy and beyond, it is also important to evaluate how maternal emotion dysregulation may affect these dynamic and reciprocal behaviors.

### 1.5. The Impact of Maternal Emotion Dysregulation on Mother–Infant Interactional Synchrony

Currently, only three studies have directly examined the relationship between maternal emotion regulation and mother–infant synchrony (Doba et al., 2022; Lotzin et al., 2015, 2016). Lotzin and colleagues (2015) evaluated maternal emotion regulation and gaze synchrony in 68 German mother–infant dyads and found a positive association between maternal emotion dysregulation and gaze synchrony between mother and infant, particularly among mothers diagnosed with a mood disorder (Lotzin et al., 2015). In another study using the same sample, Lotzin and colleagues evaluated the impact of maternal emotion regulation specifically on facial affect synchrony between mother and infant (Lotzin et al., 2016). Their findings revealed a positive association between maternal emotion dysregulation and mother–infant facial affect synchrony, with greater maternal emotion dysregulation related to greater synchrony in mothers diagnosed with a mood disorder. Additionally, a study by Doba and colleagues (2022) evaluated maternal emotion dysregulation and mother–infant synchrony with 72 French mother–infant dyads. Synchrony in this study was assessed through gaze, verbal, and motor synchrony. The findings showed that when infants were exposed to stress (i.e., the Strange Situation task), maternal emotion dysregulation led to higher symptoms of maternal anxiety, which, in turn, were associated with heightened synchrony across all three domains (Doba et al., 2022).

While the literature examining direct associations between maternal emotion dysregulation and mother–child interactional synchrony is scarce, several studies have identified impacted interactional synchrony in mother–infant dyads that have mothers with psychological conditions associated with emotion dysregulation (e.g., depression, anxiety; Field et al., 1989; Field et al., 1990; Kaitz et al., 2010; Moore et al., 2016). However, there have been discrepancies in the directionality of the effect observed in these studies. Studies that directly evaluate the effect of maternal emotion dysregulation on mother–infant synchrony generally report a positive association (Doba et al., 2022; Lotzin et al., 2015, 2016). By contrast, studies evaluating samples of mothers likely to be at greater risk of emotion dysregulation generally report mixed results regarding this relationship, with greater maternal psychopathology linked to less interactional synchrony between mothers and infants in some samples (Field et al., 1989; Kaitz et al., 2010; Moore et al., 2016) and greater mother–infant interactional synchrony in others (Field et al., 1990). Similarly, both less interactional synchrony and greater interactional synchrony have been linked to maladaptive outcomes in infant development (Beebe et al., 2010; Lotzin et al., 2015). Such uncertainties underscore the need to continue evaluating how maternal emotion dysregulation may influence the mother–infant bond.

To date, no studies have evaluated the impact of maternal emotion dysregulation on mother–child interactional synchrony across multiple time points in non-clinical samples of mothers, without an additional stressor. Moreover, no existing studies have evaluated the relationship between maternal emotion dysregulation and mother–infant synchrony in the typical home setting.

### 1.6. Current Study

Infancy is a period of rapid developmental change, and the mother–infant relationship is important for infant development (Feldman, 2012b; Hodel, 2018). Moreover, the literature suggests that interactional synchrony between the mother and infant is one way they form closeness with important implications for infants’ social, cognitive, and behavioral development and later functioning (Feldman, 2007; Harrist et al., 1994; Niedźwiecka et al., 2018). Despite extensive literature on the implications of interactional synchrony for infant and child development, little research has examined how it develops over time during the early months of infancy (Feldman, 2012b). This is likely due, in part, to several factors, including the diverse ways in which mother–infant interactional synchrony has been defined and operationalized, a lack of focused research on specific synchronous behaviors, and limited longitudinal research methodologies. Evaluating the development of several behavioral indicators of synchrony over time may fill the sizable gap in understanding how this relationship shifts during early development.

Furthermore, maternal emotion dysregulation may adversely affect the synchrony between mother and infant (Doba et al., 2022; Lotzin et al., 2015, 2016). Mothers often experience new or worsening emotional changes that may lead to emotion dysregulation due to new life stressors and biological changes (Bjelica et al., 2018; Li et al., 2020). However, research in maternal emotion dysregulation and interactional synchrony has been limited.

The goal of the current study is two-fold. First, the current project will contribute to the limited literature on the development of mother–infant interactional synchrony during the critical months of infancy in a non-clinical sample in a home-based setting. Second, the current project will offer insight into the relationship between maternal emotion dysregulation and mother–infant synchrony over time, across several synchrony markers. To our knowledge, this is the first study to evaluate the longitudinal impact of maternal emotion dysregulation on synchrony. Indeed, notable disagreement regarding the definition and methods for evaluating interactional synchrony remains evident. In the context of the present study, mother–infant interactional synchrony is operationalized as the frequency of coordinated behavioral engagement across several specific interconnected, rather than independent, behavioral markers (De Mendonça et al., 2015).

In considering all relevant literature as it pertains to the current study, the following aims and hypotheses are posed:

**Aim 1:** Evaluate whether mother–infant interactional synchrony changes between 4 and 6 months of age. It was hypothesized that overall synchrony between mother and infant would increase from 4 to 6 months of age.

**Aim 2:** Evaluate the relationship between maternal emotion dysregulation and mother–infant interactional synchrony when the infant is 4 months old. It was hypothesized that greater maternal emotion dysregulation would be associated with less overall synchrony at 4 months of age.

**Aim 3:** Evaluate the relationship between maternal emotion dysregulation and mother–infant interactional synchrony in mothers and their infants over time. It was hypothesized that greater maternal emotion dysregulation when the infant is 4 months old would predict less overall synchrony between mother and infant when the infant is 6 months old.

## 2. Materials and Methods

### 2.1. Participants

Participants were 90 mother–infant dyads recruited by medical professionals from several medical facilities in the Midwest, where the mothers received pregnancy-related care. Mothers were eligible to participate in the study if they met the following criteria: (1) speak and understand English, (2) are over the age of 18 years, (3) delivered a baby vaginally, (4) gave birth no more than eight weeks prior, and (5) there were no mother–infant care interruptions, such that the baby did not return home from the hospital with the mother.

Participant demographics for the mothers are described in Table 1. Most mothers were white, employed full-time, and came from a family with a gross annual income of over $100,000. For most mothers, this was not their first pregnancy. Infants were born between October of 2023 and March of 2025. Of the infants, 40% were male and 60% were female. According to their mothers, 32% of the infants were born early, 50% were born on time, and 18% were born later than expected.

### 2.2. Procedure

This study was part of a larger project that collected data from mothers and their infants over three time points (2 months, 4 months, and 6 months). Mothers were provided with study information and either contacted study personnel or signed up via a QR code on a flyer to express interest in participating. This occurred either during the mother’s delivery visit (before discharge) or during a follow-up care appointment.

Mother–infant interactional synchrony was evaluated at two time points: at 4 and 6 months of age. Before each visit, mothers were asked to complete a Qualtrics survey that included a questionnaire assessing emotion dysregulation. For the 4-month and 6-month visit, mothers were encouraged to: (1) schedule the session when the infant was most likely to be rested and fed; (2) use a comfortable spot on the floor; (3) limit outside distractions; and (4) bring three objects the infant enjoys. All study visits were conducted via Zoom and recorded.

During each visit, time was spent building rapport and orienting mothers to the session. Mother–infant interactional synchrony data were collected via a 10-min free-play behavioral observation of the mother and infant. Before the session, mothers were instructed on the optimal positioning for themselves and the infant to ensure both were within the video camera’s frame, thereby providing the best view for coding. During the 10-min free-play exercise, the mother was instructed to play with the infant as she normally would. Video recordings were later used for behavioral observation coding.

### 2.3. Measures

#### 2.3.1. Demographics

Information on the mother’s age, income, race/ethnicity, employment status, and total number of births was obtained. The infant’s sex and timing of delivery were also obtained.

#### 2.3.2. Maternal Emotion Dysregulation

The Difficulties in Emotion Regulation Scale—Short Form (DERS-SF) is an 18-item self-report measure used to assess emotion regulation difficulties (Kaufman et al., 2016). The DERS-SF evaluates the frequency with which respondents experience a variety of feelings or behaviors related to emotion regulation or dysregulation. Responses are recorded on a 5-point Likert scale with higher scores indicating greater emotion dysregulation. Cronbach’s alpha for the current study was .82 at 4 months and .81 at 6 months.

#### 2.3.3. Mother–Infant Interactional Synchrony

Synchrony between mother and infant was evaluated using the Taxonomy of Interactional Synchrony (TIS; De Mendonça et al., 2015). The TIS assesses mother–infant synchrony across the following domains: physical proximity, visual orientation, upper-body orientation, and dyadic involvement. Physical proximity, visual orientation, and upper-body orientation are “microanalytic,” and each behavior contributes to the synchronous interaction between mother and infant. Specifically, dyadic behaviors evaluated with the TIS are coded on a 4-point scale, with higher scores indicating greater synchrony between mother and infant (De Mendonça et al., 2011). The TIS offers a uniquely “multidimensional” approach to evaluating interactional synchrony that emphasizes the complementary, rather than independent, behavioral markers that characterize interactional synchrony.

**Physical Proximity.** Physical proximity refers to the distance between the mother and infant. Physical proximity coding was slightly modified from the original taxonomy (which used the metric system) to be adapted for U.S. coders and the developmental levels of this study’s infant participants. The adapted coding system for physical proximity included the following codes: 1 = *More than 2 ft*; 2 = *Between 1 and 2 ft*; 3 = *Less than 1 ft, but the social partners do not touch*; and 4 = *The social partners touch each other*. A physical proximity code was assigned based on the distance between the mother and infant on the final frame of each 10-s interaction.

**Visual Orientation.** Visual orientation (i.e., whether the mother and infant are looking in the same direction) is a micro-analytic behavior coded using the following codes from TIS: 1 = *The social partners look in different directions*; 2 = *Unilateral, with the child looking at the parent or the parent looking at the child*; 3 = *The social partners look in the same direction*; and 4 = *The social partners look at each other*. A visual orientation code was assigned based on the mother and infant’s gaze orientation on the final frame of each 10-s interaction.

**Upper Body Orientation.** Upper-body orientation (i.e., whether the mother and infant face each other) is a micro-analytic behavior coded using the following TIS codes: 1 = *The social partners face different directions*; 2 = *Unilateral, with the child facing the parent or the parent facing the child*; 3 = *The social partners face in the same direction*; and 4 = *The social partners face each other*. An upper-body orientation code was assigned based on how the mother and infant were facing on the final frame of each 10-s interaction.

**Dyadic Involvement.** Unlike the other codes, dyadic involvement is not microanalytic; it reflects the degree of joint engagement in a shared activity. Dyadic involvement was coded using the following TIS codes: 1 = *No involvement*; 2 = *Unilateral behavior (initiation of a gesture or talk with no response)*; 3 = *Joint attention (involving attention but no manipulation)*; and 4 = *Joint activity (involving manipulation of the same object)*. Joint activity can be characterized by mutual engagement in dialogue, mutual manipulation or play with an object, or other mutually engaged behaviors. Dyadic involvement was coded based on the longest instance of mutual engagement between mother and infant within a 10 s interaction and was coded every 10-s.

**Overall Synchrony.** While not an explicit measurement marker included in the TIS, the present study offers a cumulative measurement of behavioral interactional synchrony, in addition to individual behavioral markers. In the context of this study, overall synchrony is represented by the total scores obtained from the individual behavioral markers evaluated, including physical proximity, visual orientation, upper-body orientation, and dyadic involvement. This combined score was used as a variable in analyses in addition to the individual markers that make up the combined score. Understanding that behavioral interactional synchrony reflects interrelated rather than independent behavioral markers, it is believed that an overall score will help to further characterize the multifaceted nature of interactional synchrony.

#### 2.3.4. Coding of Interactional Synchrony

Behavioral observations between the mother and infant lasted approximately 10 min. The first 2 min were not coded to allow time for the dyad to warm up. The final 8 min were coded in 10-s increments. Micro-analytic behaviors, including physical proximity, visual orientation, and upper-body orientation, were coded based on the final frame of each 10-s observation. Dyadic involvement was coded every 10 s based on the entire 10-s interaction. Behavioral observations were coded using Behavioral Observation Research Interactive Software (BORIS), a program that allows videos to be uploaded and complex moment-to-moment behavioral observations to be micro-analyzed and coded (Friard & Gamba, 2016).

The coding of all mother–infant interactions was carried out by the first author and one research assistant. A lengthy training process was undertaken prior to coding. To ensure coding consistency, 20% of the sessions were independently coded by the two coders and then were compared to assess interrater reliability. Inter-rater reliability, using Cohen’s kappa, for each dimension of interactional synchrony was .83 for physical proximity, .70 for visual orientation, .79 for upper-body orientation, and .67 for dyadic involvement.

### 2.4. Statistical Analyses

Data cleaning, analysis, and interpretation were conducted using the Statistical Package for the Social Sciences version 30 (SPSS v.30). Overall, 99 mother–infant dyads were enrolled in the study. Of these dyads, nine withdrew before data collection. Thus, 90 dyads provided 4-month data, and all were included in the analyses specific to that time point. However, four dyads withdrew before the 6-month data collection. Thus, 86 dyads provided 6-month data. For that reason, longitudinal analyses only used 86 dyads. No other dyads were excluded from the study. Additionally, there were no missing data in the measures used for this study.

Data were checked for statistical assumptions, including visual inspection of histograms and Shapiro–Wilk tests of normality, outliers, multicollinearity, and residual scatterplots for heteroscedasticity. Aim 1 used paired samples t-tests to evaluate the changes in synchrony indicators from when the infant was 4 months old to 6 months old. Aim 2 used linear regression to evaluate the impact of maternal emotion dysregulation on interactional synchrony indicators at 4 months. Aim 3 used linear regression to evaluate the impact of maternal emotion dysregulation at 4 months on interactional synchrony indicators at 6 months.

## 3. Results

### 3.1. Descriptive Statistics

Correlations, means, and standard deviations of all target variables in the present study are presented in Table 2. In this non-clinical sample, maternal emotion dysregulation at 4 months (*M* = 32.86, *SD* = 9.55) and at 6 months (*M* = 31.61, *SD* = 9.08) was notably lower than that reported in other non-clinical samples of women (Gouveia et al., 2022). In other words, mothers in this sample reported better emotion-regulation abilities than other non-clinical samples.

Across timeframes, dyadic involvement at 4 months was positively correlated with dyadic involvement at 6 months (*r* = .24, *p* = .03). Physical proximity at 4 months was positively correlated with physical proximity at 6 months (*r* = .24, *p* = .03). Similarly, maternal emotion dysregulation at 4 months was strongly positively correlated with emotion dysregulation at 6 months (*r* = .74, *p* < .001). Within timeframes, overall synchrony at 4 months was positively correlated with 4-month physical proximity (moderate strength; *r* = .45, *p* < .001), visual orientation (moderate strength; *r* = .54, *p* < .001), upper-body orientation (strong; *r* = .64, *p* < .001), and dyadic involvement (moderate strength; *r* = .53, *p* < .001). Similarly, overall synchrony at 6 months was positively correlated with 6-month physical proximity (moderate strength; *r* = .53, *p* < .001), visual orientation (strong strength; *r* = .75, *p* < .001), upper-body orientation (strong strength; *r* = .67, *p* < .001), and dyadic involvement (moderate strength; *r* = .52, *p* < .001). Physical proximity at 4 months was positively correlated with upper-body orientation at 4 months (*r* = .30, *p* = .01). Visual orientation at 4 months was moderately positively correlated with dyadic involvement at 4 months (*r* = .48, *p* < .001). Lastly, visual orientation at 6 months was moderately positively correlated with dyadic involvement (*r* = .45, *p* < .001) and positively correlated with upper-body orientation (*r* = .25, *p* = .02) at 6 months.

### 3.2. Changes in Mother–Infant Interactional Synchrony

The purpose of Aim 1 was to evaluate whether there were changes in mother–infant synchrony between 4 and 6 months of age. It was hypothesized that overall mother–infant synchrony would increase from 4 to 6 months of age. Changes in mother–infant synchrony were also observed for the following micro-analytic behaviors: physical proximity, visual orientation, and upper-body orientation.

Regarding overall synchrony, mother–infant interactional synchrony increased from 4 to 6 months of age. This finding supports the hypothesis posed in this study. Regarding physical proximity synchrony, no change was observed in mother–infant physical proximity between 4 and 6 months of age. Regarding the micro-analytic behavior of visual orientation, no change was observed in mother–infant visual orientation from 4 to 6 months of age. Regarding micro-analytic behavior of upper-body orientation, mother–infant upper-body orientation increased from 4 to 6 months of age. Regarding mother–infant dyadic involvement, an increase from 4 to 6 months of age was observed. Results for Aim 1 are outlined in Table 3.

### 3.3. Maternal Emotion Dysregulation and Interactional Synchrony

The purpose of Aim 2 was to evaluate the relationship between maternal emotion dysregulation and mother–infant synchrony at 4 months of age. Specifically, maternal emotion dysregulation was evaluated in relation to interactional synchrony characterized by (1) overall synchrony, (2) micro-analytic markers of synchrony, including physical proximity, visual orientation, and upper-body orientation, and (3) dyadic involvement.

Concerning maternal emotion dysregulation and overall synchrony at 4 months, the linear regression model was not statistically significant, *F*(1, 88) = 0.12, *p* = .733. Maternal emotion dysregulation at 4 months explained no variance in overall mother–infant interactional synchrony (*R*^2^ = .00). Regarding maternal emotion dysregulation and the micro-analytic behavior of physical proximity at 4 months, the linear regression model was not statistically significant, *F*(1, 88) = 1.09, *p* = .30. Maternal emotion dysregulation at 4 months explained no variance in physical proximity at 4 months (*R*^2^ = .01). In relation to maternal emotion dysregulation and the micro-analytic behavior of visual orientation at 4 months, results from the linear regression model were not statistically significant, *F*(1, 88) = 0.03, *p* = .86. Maternal emotion dysregulation at 4 months explained no variance in mother–infant visual orientation at 4 months (*R*^2^ = .00). Regarding maternal emotion dysregulation and the micro-analytic behavior of upper-body orientation at 4 months, the results from the linear regression model were not statistically significant, *F*(1, 88) = 0.12, *p* = .73. Maternal emotion dysregulation at 4 months explained no variance in mother–infant upper-body orientation at 4 months (*R*^2^ = .00). Regarding maternal emotion dysregulation and mother–infant dyadic involvement at 4 months, the linear regression model was not statistically significant, *F*(1, 88) = 0.12, *p* = .73. Maternal emotion dysregulation at 4 months accounted for no variance in mother–infant dyadic involvement at 4 months (*R*^2^ = .00).

Results for Aim 2 are displayed in Table 4. Maternal emotional dysregulation at 4 months was not associated with any 4-month marker of interactional synchrony, including overall synchrony, physical proximity, visual orientation, upper body orientation, and dyadic involvement. Thus, findings did not support the hypothesis posed in the current study.

### 3.4. Maternal Emotion Dysregulation and Synchrony over Time

The purpose of Aim 3 was to evaluate the impact of 4-month maternal emotion dysregulation on synchrony markers (overall synchrony, physical proximity, visual orientation, upper body orientation, and dyadic involvement) at 6 months. See Table 5 for the results for Aim 3.

Concerning overall synchrony, the results from the linear regression model were not statistically significant, *F*(1, 84) = 0.03, *p* = .86. Maternal emotion dysregulation at 4 months explained no variance in overall synchrony at 6 months (*R*^2^ = .00). In other words, maternal emotion dysregulation at 4 months was not a significant predictor of overall synchrony over time.

Regarding physical proximity, the results from the linear regression were not statistically significant, *F*(1, 84) = 0.00, *p* = .967. Maternal emotion dysregulation at 4 months explained no variance in physical proximity synchrony at 6 months (*R*^2^ = .00). Regarding visual orientation, the results from the linear regression model were not statistically significant, *F*(1, 84) = 0.21, *p* = .65. Maternal emotion dysregulation at 4 months explained no variance in visual orientation at 6 months (*R*^2^ = .00). Regarding upper-body orientation, the results from the linear regression model were not statistically significant, *F*(1, 84) = 0.00, *p* = .95. Maternal emotion dysregulation at 4 months explained no variance in upper-body orientation at 6 months (*R*^2^ = .00). Regarding dyadic involvement, the results from the linear regression model were not statistically significant, *F*(1, 84) = 0.02, *p* = .90. Maternal emotion dysregulation at 4 months explained no variance in dyadic involvement at 6 months (*R*^2^ = .00). Thus, maternal emotion dysregulation at 4 months did not predict any marker of synchrony over time. The hypothesis posed in the current study was not supported.

## 4. Discussion

Interactional synchrony is a fundamental component of early parent–child interactions and may serve as the building blocks for the development of attachment between mother and infant, with several implications for the infant’s later functioning (Feldman, 2007; Harrist et al., 1994; Niedźwiecka et al., 2018). Infancy is a period of rapid development for both infants and their relationships with caregivers (Feldman, 2012b; Hodel, 2018). Despite the importance of mother–infant synchrony, little research has evaluated the unique developmental trajectories of interactional synchrony. Early motherhood is vulnerable to challenges with emotional dysregulation; however, links between maternal emotional functioning and interactional synchrony have been inconsistent (Bjelica et al., 2018; Doba et al., 2022; Li et al., 2020; Lotzin et al., 2015, 2016). The present study aimed to contribute to the limited understanding of the developmental process of mother–infant interactional synchrony and inform current understandings of the relationship between maternal emotion dysregulation and mother–infant interactional synchrony over time.

### 4.1. Changes in Mother–Infant Interactional Synchrony Between 4 and 6 Months of Age

The sample in this study demonstrated increases in overall synchrony between mother–infant dyads from 4 to 6 months of age, consistent with study hypotheses. Specifically, dyadic involvement and upper body orientation increased from 4 to 6 months. There was no change in physical proximity and visual orientation during this time period.

To our knowledge, this finding represents the first documented snapshot of the developmental changes of mother–infant dyadic involvement. With increasing interaction between mothers and infants as infants age, a corresponding developmental trajectory in the degree of mutual engagement in activities is expected. The observed increase in overall interactional synchrony and upper-body orientation from 4 to 6 months of age can also be explained by this rationale. Increases in these reciprocal behaviors as the infant ages may serve as a foundation for later attachment development (Baker & McGrath, 2011). These changes over time in synchrony markers are congruent with the view of a sensitive period in which infants are drawn to socially relevant stimuli (Freeman, 2026).

Regarding physical proximity and visual orientation, no change was observed from 4 to 6 months. In considering overall infant development, it might be expected that infants’ visual orientation and physical proximity to their caregiver will decrease over time as their interest in exploring the world around them increases (Chen et al., 2023; Rheingold & Eckerman, 1970). However, attachment formation processes would also suggest greater attunement to a preferential attachment figure during sensitive periods of filial bonding (Freeman, 2026). Further research is needed to further understand how these constructs develop over time during distinct periods of infant development.

### 4.2. The Impact of Maternal Emotion Dysregulation on Interactional Synchrony

Maternal emotion dysregulation at 4 months of age was not related to concurrent overall synchrony, physical proximity, visual orientation, upper body orientation, or dyadic involvement, contrary to hypotheses. It was also hypothesized that greater maternal emotion dysregulation at 4 months would predict less overall synchrony, physical proximity, visual orientation, upper-body orientation, and dyadic involvement at 6 months. Contrary to hypotheses, maternal emotion dysregulation at 4 months did not predict overall synchrony, physical proximity, visual orientation, upper-body orientation, or dyadic involvement at 6 months. Thus, this study did not find maternal emotion dysregulation to be related to any marker of synchrony, cross-sectionally or longitudinally. However, it is important to note that this sample had low levels of emotional dysregulation and it is possible that different findings may have emerged in a higher risk sample.

This study used a variety of markers of interactional synchrony compared to prior studies that have examined the relationship between maternal emotional dysregulation and mother–infant synchrony (Doba et al., 2022; Lotzin et al., 2015, 2016). However, visual orientation was a similar marker of synchrony shared between this study and other research (Doba et al., 2022; Lotzin et al., 2015). Regarding visual orientation synchrony, the results of this study were inconsistent with those reported by Lotzin et al. (2015) and Doba et al. (2022), as maternal emotion dysregulation did not affect mother–infant visual orientation synchrony in this sample. However, the other studies evaluated dyadic interactions with elevated symptomatology (e.g., mothers with mood disorders, infants exposed to a stressor), whereas this study was conducted with a sample that had better emotion regulation functioning compared to other non-clinical samples (Gouveia et al., 2022). Thus, the lack of an association between maternal emotional dysregulation and markers of interactional synchrony in this study could be due to a restricted range of emotional dysregulation in this sample. Furthermore, other findings to date on the relationship between emotional difficulties in mothers and synchrony have been inconsistent (Beebe et al., 2010; Field et al., 1989, 1990; Kaitz et al., 2010; Moore et al., 2016). It may be that these associations are influenced by a myriad of factors, including the developmental timing and context of the mother–infant interaction, highlighting the need for further research to elucidate these issues.

Although there is some evidence suggesting that infants are uniquely attuned to their caregivers’ emotional states (Cassidy, 1994; Edwards et al., 2017) and that maternal emotion dysregulation has a measurable impact on the mother–infant relationship (Binion & Zalewski, 2018; Edwards et al., 2017; Morelen et al., 2014; Oddo et al., 2022), it may be the case that the impacts of maternal functioning that are influential in other aspects of infant development are not as relevant for synchrony during this timeframe. The lack of impact that maternal emotional dysregulation had on several markers of synchrony in this study is in concordance with the argument that infant predisposition to attach is a robust phenomenon that can occur across a variety of caregiving contexts (Freeman, 2024). Freeman (2026) argues for a conceptualization of attachment formation as a selection process operating within a sensitive period, meaning that these infant behaviors may not be dependent on caregiver functioning. This possible interpretation of the results would suggest that mother–infant synchrony may be less vulnerable to maternal dysregulation than previously expected.

### 4.3. Limitations and Future Directions

While the present study is novel in its approach to evaluating interactional synchrony over time and how maternal emotion dysregulation may affect synchrony, several limitations should be noted. Although the utility of behavioral observation methodologies lies in the ability to provide data that reflect behaviors occurring within typical interactions, it is well documented that participants who know they are being observed may modify their behavior accordingly (Bakeman & Haynes, 2015). Although it is difficult to quantify the extent to which the mother–infant interactions observed in this study deviated from typical patterns, social desirability may be a particularly relevant factor among new mothers, who experience notable societally maintained stress related to how new mothers should behave and parent (Lazarus & Rossouw, 2015).

It is also important to note that mothers in this sample reported remarkably better emotion regulation abilities compared to other non-clinical samples (Gouveia et al., 2022); study findings should be interpreted with this caveat in mind. It may be the case that mothers with greater emotion dysregulation during the early postpartum period were less likely to enroll in the present study. It would be important to determine how these findings regarding the relationship between maternal emotion dysregulation and interactional synchrony may differ in more impaired samples. Future research may seek out mother–infant dyads in which the mother is diagnosed with a psychiatric disorder associated with emotion dysregulation to observe this relationship more clearly or specifically recruit a sample facing greater systemic challenges (e.g., low-income mothers). Further, the sample size for this study was relatively small; a larger sample would allow for more complex and nuanced statistical investigation into these relationships. Additionally, the majority of this sample consisted of white, employed, high-income mothers, which may partially explain the better emotion regulation in this sample. It is crucial that future researchers study these relationships in diverse samples, to determine if the factors influencing mother–infant interactional synchrony change in other contexts.

### 4.4. Conclusions

The importance of mother–infant synchrony for infant development is well established (Bell, 2020; Black & Logan, 1995; Feldman et al., 1996; Harrist et al., 1994; Isabella & Belsky, 1991; Leclère et al., 2014; MacLean et al., 2014; Niedźwiecka et al., 2018; Rocissano et al., 1987). However, little is known about how mother–infant interactional synchrony manifests and transforms during a period of rapid developmental change in infancy (Feldman, 2012b). When mothers experience heightened emotions (Bjelica et al., 2018; Li et al., 2020), likely intensified by biological, psychological, and social factors associated with the postpartum period, it is important to understand how mothers’ emotion regulation abilities might influence interactional synchrony between mothers and infants. Yet, limited research, often with inconsistent results, has been conducted in this area, highlighting the need for more focused studies. The current study aimed to examine developmental changes in several indicators of mother–infant synchrony and their relationship with maternal emotional dysregulation throughout key months of infant development.

Results from this study revealed developmental increases in overall synchrony, dyadic involvement, and upper-body orientation, but not in physical proximity and visual orientation between 4 and 6 months of age. These findings add to the scarce literature on the development of interactional synchrony during the formative early years of development. Moreover, the lack of associations found between maternal emotion dysregulation and interactional synchrony aligns with emerging frameworks of attachment proposing that filial synchrony may withstand fluctuations in maternal functioning (Freeman, 2026).

It is clear that the processes involved in infant development and maternal bonding are complex and multifaceted, highlighting the need for further study in this important area. Understanding how synchrony markers change across infant development has practical implications for developing screening methodologies for infants that may be at risk for disrupted bonding, as assessment of infants is inherently challenging and synchrony markers may provide an observable indicator of the infant’s functioning. Understanding how various factors, such as maternal emotional dysregulation, may influence synchrony has important clinical implications for promoting healthy infant development.

## Figures and Tables

**Table 1:** Maternal Demographics of Study Sample.

Characteristics	%	*n*
Age		
Range	20–40	
*M* (*SD*)	29.86 (4.42)	
Race/Ethnicity		
Hispanic	8%	7
White alone, non-Hispanic	83%	75
Black or African American alone, non-Hispanic	1%	1
American Indian or Alaska Native, non-Hispanic	3%	3
Asian alone, non-Hispanic	1%	1
Multiracial, non-Hispanic	4%	3
Employment status		
Employed for salary/wage and working 40 or more hours per week	60%	54
Employed for salary/wage and working 1–39 h per week	18%	16
Not employed, looking for work	1%	1
Not employed, not looking for work	17%	15
Self-employed	3%	3
Student	1%	1
Family gross annual income (pre-tax)		
Less than $25,000	3%	3
$25,000–$50,000	3%	3
$50,000–$75,000	18%	16
$75,000–$100,000	22%	20
More than $100,000	54%	48
Total # of deliveries (vaginal or cesarean)		
1 delivery	37%	33
2 deliveries	34%	31
3 deliveries	17%	15
4 deliveries	8%	7
5+ deliveries	4%	4

**Table 2:** Means, Standard Deviations, and Correlations for Primary Variables of Interest.

Variable	*M* (*SD*)	1	2	3	4	5	6	7	8	9	10	11
1. 4-Month Maternal ED	32.86 (9.55)	--										
2. 4-Month Overall Synchrony	565.04 (38.41)	.04	--									
3. 4-Month Physical Proximity	180.16 (12.85)	.11	.45 **	--								
4. 4-Month Visual Orientation	123.39 (16.99)	.02	.54 **	−.05	--							
5. 4-Month Upper Body Orientation	129.32 (23.92)	−.04	.64 **	.30 **	−.12	--						
6. 4-Month Dyadic Involvement	132.18 (14.92)	.04	.53 **	−.05	.48 **	−.09	--					
7. 6-Month Maternal ED	31.61 (9.08)	.74 **	−.05	.07	−.04	−.03	−.08	--				
8. 6-Month Overall Synchrony	581.49 (35.33)	−.02	.19	.09	.14	.06	.16	−.08	--			
9. 6-Month Physical Proximity	180.06 (12.27)	.01	.16	.24 *	−.03	.10	.08	.07	.53 **	--		
10. 6-Month Visual Orientation	121.12 (14.85)	−.05	.11	.07	.11	−.01	.10	−.10	.75 **	.17	--	
11. 6-Month Upper Body Orientation	140.87 (17.78)	.01	.12	−.02	.16	.07	.03	−.04	.67 **	.19	.25 *	--
12. 6-Month Dyadic Involvement	139.44 (11.07)	−.01	.10	−.03	.07	−.02	.24 *	−.13	.52 **	.07	.45 **	−.01

*Notes.* ED = Emotion dysregulation. * *p* < .05, ** *p* < .001.

**Table 3:** Changes in Interactional Synchrony From 4- to 6-Months.

	4-Month *M* (*SD*)	6-Month *M* (*SD*)	*t*(85)	*p*	Cohen’s *d*	95% C.I.	
						LL	UL
Overall Synchrony	565.59 (*38.49*)	581.49 (*35.33*)	−3.14	.002	−.34	−.56	−.12
Physical Proximity	179.66 (*12.93*)	180.06 (*12.27*)	−0.24	.820	−.03	−.24	.19
Visual Orientation	123.65 (*1.83*)	121.12 (*1.60*)	1.11	.272	.12	−.09	.33
Upper Body Orientation	129.30 (*24.45*)	140.87 (*17.78*)	−3.68	<.001	−.40	−.62	−.18
Dyadic Involvement	132.94 (*14.21*)	139.44 (*11.08*)	−3.11	<.001	−.41	−.62	−.19

*Note*. LL = Lower Limit; UL = Upper Limit.

**Table 4:** Associations between Maternal Emotion Dysregulation and Interactional Synchrony at 4-Months.

Variable	*B*	*SE*	*β*	*t*	*p*	*R* ^2^
**4-Month Overall Synchrony**						
4-Month Maternal Emotion Dysregulation	.15	.43	.04	.34	.73	.00
**4-Month Physical Proximity**						
4-Month Maternal Emotion Dysregulation	.15	.14	.11	.05	.30	.01
**4-Month Visual Orientation**						
4-Month Maternal Emotion Dysregulation	.03	.19	.02	.17	.86	.00
**4-Month Upper Body Orientation**						
4-Month Maternal Emotion Dysregulation	−.09	.27	−.04	−.35	.73	.00
**4-Month Dyadic Involvement**						
4-Month Maternal Emotion Dysregulation	.06	.17	.04	.35	.73	.00

**Table 5:** The Impact of Maternal Emotion Dysregulation on Mother-Infant Interactional Synchrony from 4-Months to 6-Months.

Variable	*B*	*SE*	*β*	*t*	*p*	*R* ^2^
**6-Month Overall Synchrony**						
4-Month Maternal Emotion Dysregulation	−.07	.40	−.02	−.18	.86	.00
**6-Month Physical Proximity**						
4-Month Maternal Emotion Dysregulation	.01	.14	.01	.04	.97	.00
**6-Month Visual Orientation**						
4-Month Maternal Emotion Dysregulation	−.08	.17	−.05	−.45	.65	.00
**6-Month Upper Body Orientation**						
4-Month Maternal Emotion Dysregulation	.01	.20	.01	.06	.95	.00
**6-Month Dyadic Involvement**						
4-Month Maternal Emotion Dysregulation	−.02	.13	−.02	−.13	.90	.00

## Data Availability

The data that support the findings of this study are available on request from the corresponding author (B.A.D.).
